# Integrated LC-MS/MS Analytical Systems and Physical Inspection for the Analysis of a Botanical Herbal Preparation

**DOI:** 10.3390/molecules200610641

**Published:** 2015-06-09

**Authors:** Kuan-Ming Lai, Yung-Yi Cheng, Tung-Hu Tsai

**Affiliations:** 1 Institute of Traditional Medicine, School of Medicine, National Yang-Ming University, No. 155, Sec. 2, Li-Nong St, Beitou District, Taipei 11221, Taiwan; E-Mails: lancaster021@hotmail.com (K.-M.L.); vininecheng@gmail.com (Y.-Y.C.); 2 Graduate Institute of Acupuncture Science, China Medical University, No. 91, Hsueh-Shih Road, Taichung 40402, Taiwan; 3 School of Pharmacy, College of Pharmacy, Kaohsiung Medical University, No. 100, Shih-Chuan 1st Road, Kaohsiung 80708, Taiwan; 4 Department of Education and Research, Taipei City Hospital, No.145, Zhengzhou Rd., Datong Dist., Taipei 10341, Taiwan

**Keywords:** Traditional Chinese Medicine, quality control, herbal analysis, traditional herbal formulation

## Abstract

The herbal decoction process is generally inconvenient and unpleasant. To avoid using herbal medicine decoctions, various high-quality industrial and pharmaceutical herbal decoction products have been used in clinical applications for more than ten years in Taiwan. However, the consistency and standardization of the quality of these herbal medicines are goals that remain to be achieved. The aim of study was to develop a validated liquid chromatography-tandem electrospray ionization mass spectrometry (LC-MS/MS) method to determine the biomarkers astragaloside I, astragaloside IV, formononetin, cinnamic acid, paeoniflorin and gingerol in the herbal preparation known as Huangqi-Guizhi-Wuwu (HGW). To investigate the physical quality of HGW, methods such as scanning electron microscopy, light microscopy with Congo red and potassium iodine staining, solubility measurements, swelling power tests, and crude fiber analysis were used to identify additives in commercial pharmaceutical products. The optimal LC-MS/MS multiple reaction-monitoring system included a gradient program using 5 mM ammonium acetate buffer with 0.05% formic acid/methanol. The results demonstrate deviations in biomarker content across different brands. In addition to the herbal extract, starch and excipients in the pharmaceutical granule, and crushed crude herb powder was added to the pharmaceutical products to increase their herbal ingredient content. In conclusion, a rigorous examination should be performed to certify the quality of the herbal products.

## 1. Introduction

Traditional Chinese Medicine is one of the most important medical sources for human medicines. The frequency and the cost of Traditional Chinese Medicine treatments have grown dramatically in recent years [1,2]. However, quality controls for these herbal products still do not demonstrate batch-to-batch production consistency even for those from the same industrial pharmaceutical company. Huangqi-Guizhi-Wuwu (HGW) decoction is a popular traditional Chinese prescription that ameliorates peripheral diseases such as diabetic peripheral neuropathy. This herbal formulation is also combined with acupuncture for the treatment of lymphedema [3]. HGW is recorded in the Synopsis of Prescriptions of the Golden Chamber (Jingui Yaolue), a classic traditional Chinese medicine treatise with ancient and contemporary case studies.

The herbal formulation HGW is made up of *Astragalus membranaceus* (Chinese herbal name: Huang-Qi), *Cinnamomum cassia* (Chinese herbal name: Gui-Zhi), *Paeonia lactiflora* (Chinese herbal name: Bai-Shao), *Zingiber officinale* (Chinese herbal name: Jiang) and *Ziziphus jujube* (Chinese herbal name: Da-Zao). The herbal formulation is derived from Gui-Zhi-Tang, a classical Traditional Chinese Medicine herbal formulation. In Gui-Zhi-Tang, *Glycyrrhiza glabra* (Chinese herbal name: Gan-Cao) is removed for its moderate effects and replaced with *Astragalus membranaceus* to modulate blood from bio-energy *Qi* to active *Yang* [4]. According to the theory of Traditional Chinese Medicine, the herbal substitute for this formulation is chosen to expel the pathogenesis of peripheral disease. Triterpene saponins and isoflavonoids are the main active components in Radix Astragali [5]. Clinical research shows that Radix Astragali can improve cardiovascular function, strengthen the immune response and enhance vitality [6]. *Cinnamomum cassia* contains vital oils and many derivatives such as cinnamaldehyde, cinnamic acid, and cinnamate, and it has been reported to have anti-inflammatory, antidiabetic, antimicrobial, anticancer and lipid-lowering properties, as well as increasing blood circulation [7]. Paeoniflorin is one of the main active ingredients that is used as a phytochemical marker for quality control, and it possesses anti-inflammatory, anticoagulant, anti-oxidant and neuroprotective effects [8]. *Zingiber officinale* is also a widely used in foods, beverages, spices and dietary supplements. Gingerol is the major pungent, nonvolatile, phenolic component of ginger. The herb *Zingiber officinale* is used to disperse bio-energy *Qi*, keep *Qi* in motion and assist in regulating blood circulation; this is the reason for the interdependence between *Yin* and *Yan*g [9]. Previous studies have revealed that *Ziziphus jujube* contains various constituents including triterpenic acids, phenolic acids, and polysaccharides, which may also possess bioactivity [10]. To provide high quality herbal medicines, most Traditional Chinese Medicine products are manufactured by industrial herbal pharmaceutical companies, and the application for clinical trials can be time-consuming in Taiwan. However, consistency and standardization for the quality of the herbal medicine are still controversial. To granulate the herbal extracts, starch and adjuvants are added during the manufacturing processes. It is also legal to add raw herbal powder and starch into the herbal pharmaceutical products to modify their content. It is important to inspect the content of Chinese herbal pharmaceutical products [11], as various additives may cause the quality of herbal pharmaceutical products to remain unknown. To address these issues, the aim of this study was to develop a method of coupled liquid chromatography-tandem electrospray ionization mass spectrometry method to determine the biomarkers astragaloside I, astragaloside IV, formononetin, cinnamic acid, gingerol and paeoniflorin for the chemical examination of Huangqi-Guizhi-Wuwu. Physical methods such as scanning electron microscopy, light microscopy of Congo red and potassium iodine staining, solubility test and crude fiber analysis were also used to examine the particle appearance, solubility and the contents of crude fibers for identifying the herbal formulation.

## 2. Results and Discussion

### 2.1. Method Development

**Figure 1:**
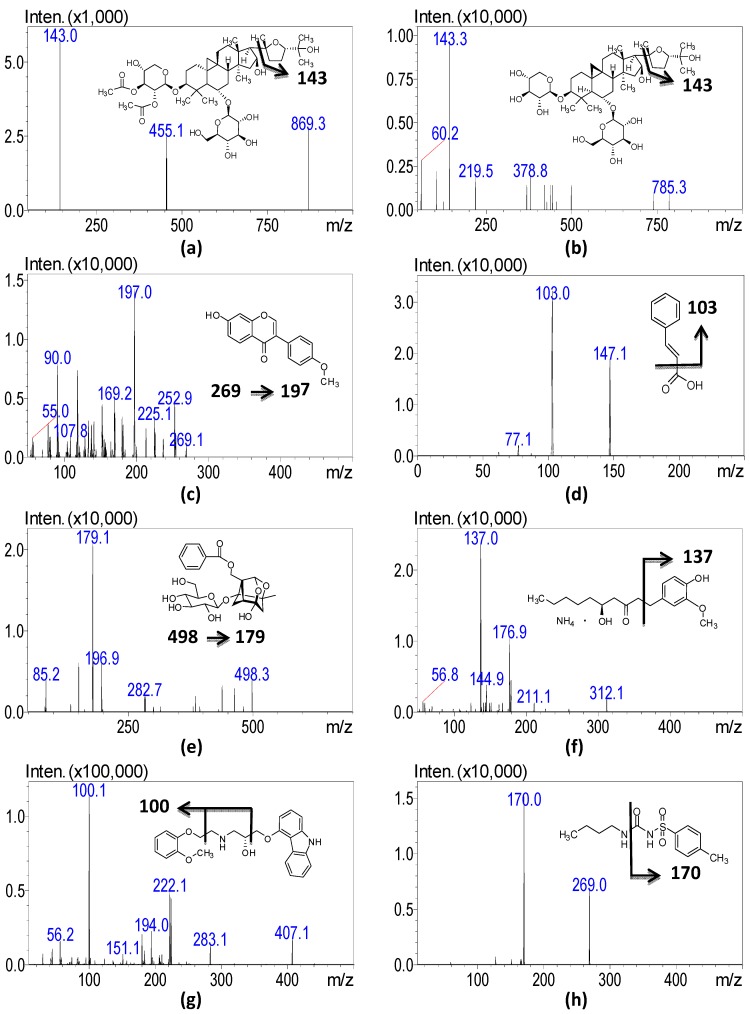
The mass spectra and structures of six active components in HGW and two internals: (**a**) astragaloside I; (**b**) astragaloside IV; (**c**) formononetin; (**d**) cinnamic acid; (**e**) paeoniflorin; (**f**) gingerol; (**g**) carvedilol and (**h**) tolbutamide.

To examine the separation of analytes on the stationary phase of the chromatographic column, different types of columns such as a Kinetex C18 column (2.1 mm × 100 mm, particle size 2.6 μm, Phenomenex, Torrance, CA, USA) and a Acquity BEH C_18_ column (2.1 mm× 100 mm, particle size 1.7 μm, Waters, Dublin, Ireland) were investigated. Both C18 columns exhibited good selectivity and sensitivity, but the retention time of the hydrophilic compounds on the Waters C18 column was longer than those on the Phenomenex Kinetex column. This result suggests that hydrophilic compounds such as cinnamic acid present a stable elution profile and less solvent interference for this analytical system. The mobile phase consisted of 5 mM ammonium acetate (mobile phase A) and 100% methanol with 0.05% formic acid (mobile phase B). Although acetonitrile provides a lower pressure profile for some compounds, we finally chose methanol as the organic solvent due to the consideration of signal high intensity. In the LC system, formic acid was added to the mobile phase to improve the ionization response and to ameliorate peak tailing. A previous report demonstrated that astragalosides are relatively stable to acids [12], but cinnamic acid shows poor sensitivity in acidified solvents, so we modified the content of formic acid to 0.05%. The mass spectra are shown in Figure 1. The positive ESI mode of [M + H]^+^ was used to yield the precursor ions of astragaloside I, astragaloside IV and formononetin. A product ion of *m/z* 143 was found in astragaloside I and astragaloside IV which having a similar cycloartane triterpene glycoside core structure. Formononetin is an isoflavone, and in Hussein’s study, it was demonstrated that the characteristic fragments of formononetin were *m/z* 253, 225, and 197 [13]. Consistent fragments of *m/z* 253 and *m*/*z* 197 were found in our results, with *m/z* 197 exhibiting high intensity. Paeoniflorin and 6-gingerol formed [M + NH_4_]^+^ precursor ions. Paeoniflorin was cleaved into *O*-glucose to produce the major product ion at *m/z* 179.2. The fragments of gingerol-related compounds such as gingerol were demonstrated at *m/z* 277, 177, and 137 [14,15].

All of the fragment ions could be detected in our study, and the product ion at *m/z* 137 was selected as having good sensitivity. The detection of cinnamic acid was performed in negative mode, and the peak at *m/z* 103 after losing a carboxylic acid moiety was obtained. The internal standards (carvedilol and tolbutamide) were analyzed in positive and negative ESI modes, respectively. Detailed mass spectrometry conditions are summarized in Table 1.

**Table 1:** The LC-MS/MS conditions for the identification of the six active components and two internal standards.

	MW ^a^	Ion Mode	RT ^b^	CE ^c^	Q1 Mass ^d^	Q3 Mass ^d^
***Astragalus membranaceus***						
Astragaloside I	868.4	+	5.8	−19	869.3 [M + H]^+^	143.0
Astragaloside IV	784.9	+	6.4	−21	785.3 [M + H]^+^	143.3
Formononetin	268.3	+	5.2	−42	269.1 [M + H]^+^	197.0
***Paeonia lactiflora***						
Paeoniflorin	480.2	+	3.6	−26	498.3 [M + NH_4_]^+^	179.1
***Zingiber officinale***						
Gingerol	294.3	+	5.2	−25	312.0 [M + NH_4_]^+^	137.2
***Cinnamomum cassia***						
Cinnamic acid	148.1	−	3.4	15	147.1 [M − H]^−^	103.0
Carvedilol (IS)	406.4	+	4.9	−35	407.1 [M + H]^+^	100.1
Tolbutamide (IS)	270.3	−	4.4	20	269.0 [M − H]^−^	170.0

**^a^** MW: molecular weight (unit: a.m.u); **^b^** RT: retention time (unit: minute); **^c^** CE: collision energy (unit: electron volt); **^d^** Theunit is *m/z*.

### 2.2. Method Validation

The calibration curves were linear over a concentration range of approximately 10–1000 ng/mL. Linear equations and the corresponding correlation coefficients (*r*^2^) were obtained as follows: y = 0.0012x + 0.009 (*r*^2^ = 0.9997, astragaloside I), y = 0.0114x + 0.029 (*r*^2^ = 0.9998, astragaloside IV), y = 0.0216x + 0.175 (*r*^2^ = 0.9998, formononetin) y = 0.0086x ‒ 0.02581 (*r*^2^ = 0.9997, cinnamic acid), y = 0.0463x + 0.4739 (*r*^2^ = 0.9998, paeoniflorin), y = 0.4899x + 1.5092 (*r*^2^ = 0.9998, gingerol), where x represents the concentration of each compound and y represents the peak area ratio. The analytical results of the intra-day and inter-day precision/accuracy for the six compounds are shown in Table 2. These results were established from three replicate runs performed at five concentrations. The inter-day and intra-day precision of the six analytes ranged from 0.07 to 13.61, while the accuracy was within ‒11.67% and 10.74%. The observed concentrations might lower or higher than the nominal concentrations due to instrumental or artificial errors. Therefore, accuracy has − values and sometimes + values. However, the precision is based on the standard deviation of the observed concentration in different runs, and its contribution to the precision is always +. The data show that the lower limits of detection (LLOD) of the analytes were 1 ng/mL (astragaloside I, astragaloside IV, gingerol and paeoniflorin) and 5 ng/mL (formononetin and cinnamic acid), respectively. The lower limits of quantitation (LLOQ) for astragaloside IV and gingerol were set at 10 ng/mL, and 25 ng/mL for astragaloside I, formononetin, cinnamic acid and paeoniflorin.

**Table 2:** Intra-day and inter-day precision and accuracy of the six active components of HGW.

Nominal Concentration (ng/mL)	Intra-Day			Inter-Day		
	Observed Concentration (ng/mL)	Accuracy (%)	Precision (%)	Observed Concentration (ng/mL)	Accuracy (%)	Precision (%)
Astragaloside I						
25	25.21 ± 1.49	0.86	5.90	23.83 ± 0.85	−4.68	3.55
50	47.68 ± 3.93	−4.64	8.24	48.78 ± 1.73	−2.44	3.56
250	259.48 ± 5.70	3.79	2.20	237.78 ± 5.93	−4.89	2.49
500	488.38 ±15.04	−2.32	3.08	512.51 ± 13.76	2.50	2.69
1000	987.19 ± 9.88	−1.28	1.00	983.27 ± 22.24	−1.67	2.26
Astragaloside IV						
10	9.23 ± 0.46	−7.72	4.98	9.82 ± 1.34	−1.84	13.61
50	23.64 ± 1.44	5.43	6.08	49.21 ± 3.29	−1.58	6.68
250	50.21 ± 1.14	0.41	2.26	250.03 ± 7.69	0.01	3.08
500	246.48 ± 8.80	−1.41	3.57	515.21 ± 5.45	3.04	1.06
1000	517.22 ± 6.27	3.44	1.21	997.18 ± 0.68	−0.28	0.07
Formononetin						
25	22.81 ± 1.33	−8.75	5.82	22.08 ± 0.47	−11.67	2.12
50	46.01 ± 1.93	−7.98	4.19	46.77 ± 2.34	−6.46	5.01
250	253.44 ± 10.43	1.38	4.11	254.42 ± 5.85	1.77	2.30
500	506.04 ± 10.04	1.21	1.98	509.04 ± 15.79	1.81	3.10
1000	995.55 ± 2.29	−0.44	0.23	994.74 ± 6.45	−0.53	0.65
Cinnamic acid						
25	27.69 ± 1.95	10.74	7.05	26.32 ± 1.53	5.29	5.83
50	53.33 ± 1.62	6.65	3.04	49.83 ± 3.40	−0.33	6.82
250	243.41 ± 5.31	−2.64	2.18	236.01 ± 11.10	−5.60	4.70
500	497.55 ± 10.83	−0.49	2.18	487.97 ± 23.74	−2.41	4.87
1000	1001.63 ± 5.97	0.16	0.60	997.47 ± 8.11	−0.25	0.81
Paeoniflorin						
25	24.99 ± 1.55	−0.04	6.21	24.50 ± 3.31	−2.01	13.50
50	49.44 ± 3.38	−1.13	6.83	51.09 ± 4.00	2.17	7.84
250	257.68 ± 16.41	3.07	6.37	245.60 ± 10.78	−1.76	4.39
500	501.05 ± 7.91	0.21	1.58	505.76 ± 1.82	1.15	0.36
1000	993.47 ± 9.92	−0.65	1.00	998.49 ± 0.90	−0.15	0.09
Gingerol						
10	9.58 ± 0.84	−4.25	8.82	9.26 ± 0.47	7.38	5.10
50	49.13 ± 1.79	−1.74	3.65	50.31 ± 2.26	0.61	4.49
250	251.93 ± 2.66	0.77	1.05	251.84 ± 7.15	0.73	2.84
500	500.08 ± 7.34	0.02	1.47	497.17 ± 8.01	−0.57	1.61
1000	999.65 ± 3.05	−0.04	0.31	1000.92 ± 2.27	0.09	0.23

Each value is expressed as the mean ± S.E.M (*n* = 3).

### 2.3. Application to the HGW Samples

The experimental results demonstrated that the herbal content levels measured for the various brands of commercial industrial pharmaceutical products did not differ significantly (Table 3). Paeoniflorin was abundant in the pharmaceutical industrial products. Triterpenoids such as astragaloside were scarce, although *Astragalus membranaceus* is the major herb in this prescription. The changes in content of many compounds in constituent herbs may be due to the origin, growth time, growing environment, processing and storage of the herbal product. In recent years, Radix Astragali has become increasingly scarce and Radix Hedysari, from the same family as Radix Astragali, has been widely used instead of Radix Astragali. Most of the crude herbs sold commercially in Taiwan are Radix Hedysari, due to the lower price. However, the content of astragaloside I in Radix Hedysari is small, and the immunological effects of the two species also show significant differences [5,16]. Therefore, we chose Radix Astragali as the ingredient in this study. The constituent 6-gingerol would be significantly converted into 6-shogaol in boiling water and under alkaline conditions. Therefore, the *Zingiber officinale* decoction methods contribute to the different compound levels. For instance, fresh *Zingiber officinale* and dried *Zingiber officinale* may have different amounts of gingerol [17]. Although the extractive preparation was controlled at the proper conditions, the content of gingerol in lab extracts is lower than in the herbal pharmaceutical products.

**Table 3:** The content of astragaloside I, astragaloside IV, formononetin, cinnamic acid, paeoniflorin, gingerol in lab-made herbal extract (EX) and five brands (A–E) of HGW herbal pharmaceutical products.

Components	EX	A	B	C	D	E
Astragaloside I	0.46 ± 0.024	0.31 ± 0.007	0.52 ± 0.005	0.81 ± 0.004	0.39 ± 0.006	0.36 ± 0.001
Astragaloside IV	0.09 ± 0.002	0.12± 0.005	0.16 ± 0.003	0.10 ± 0.003	0.08± 0.002	0.07 ± 0.001
Formononetin	0.83 ± 0.021	0.31 ± 0.004	0.68 ± 0.010	0.46 ± 0.006	0.54 ± 0.006	0.34 ± 0.002
Cinnamic acid	0.24 ± 0.010	0.32 ± 0.009	0.34 ± 0.005	0.19 ± 0.006	0.31 ± 0.007	0.17 ± 0.004
Paeoniflorin	4.15 ± 0.044	2.26 ± 0.013	3.72 ± 0.003	2.09 ± 0.047	2.97 ± 0.002	1.67 ± 0.016
Gingerol	0.08 ± 0.003	0.91 ± 0.006	0.25 ± 0.007	0.36 ± 0.010	0.30 ± 0.007	0.80 ± 0.001

Each value is expressed as the mean ± S.E.M (*n* = 3) and the unit is mg/g.

### 2.4. Physical Examination of the Pharmaceutical Additives

To evaluate the starch granules and herbal powders of the pharmaceutical products, a series of physical examinations were performed. Based on the previous analysis, data analysis can be a rapid and reliable indicator for quality control and for determining the addition of raw herbal powders and starch to HQW [18]. The properties of the crude drugs of HQW were examined by extracting, purifying, lyophilizing, and granulating in different proportions crude drugs (g), extracts (g) and starch granulations (g) as: A (6.2:1:0.67), B (6.2:1:1), C (4.12:1:0.5), D (7:1:0.5), E (6.22:1:0.67). In particular, the package insert of brand C indicates that 4.2 g of microcrystalline cellulose is additionally added to every 14.4 g of pharmaceutical powder. The associated properties are determined by methods such as scanning electron microscopes, Congo red staining, solubility and sedimentation of swollen granules. All these methods measure slightly different physical properties and have unique and inherent advantages.

#### 2.4.1. Scanning Electron Microscopy

**Figure 2:**
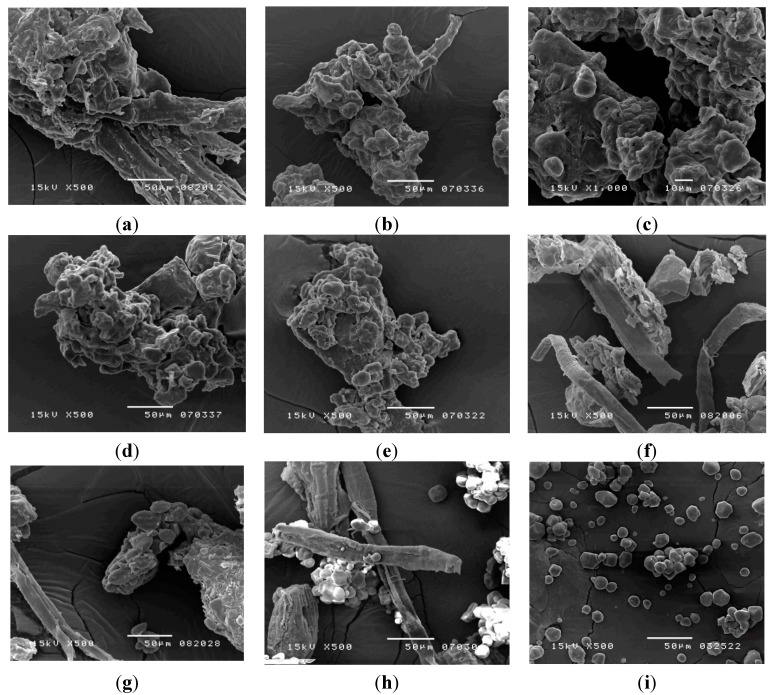
Scanning electron microscope images: (**a**) brand A; (**b**) brand B; (**c**) brand C; (**d**) brand D; (**e**) brand E; (**f**) herbal powder; (**g**) herbal powder:brand A = 1:1; (**h**) corn starch:herbal powder = 1:1 and (**i**) corn starch.

To investigate the outer appearance and particle size of the herbal powder, a scanning electron microscope (SEM) was used. The data demonstrated that the herbal pharmaceutical products appeared irregular in particle-merged shape due to gelatinization during the manufacturing process (Figure 2). Compared with the herbal pharmaceutical products, the starch particles (Figure 2i) were granular and pelletized. Analysis of the raw herbal powder showed that the crushed botanical fibers appeared stripy, elongated and filamentous, as depicted in the photograph of raw herbal powder (Figure 2f). These results demonstrated that the herbal pharmaceutical products of HQW (Figure 2a–e) could be distinguished by the presence of the additives of starch and raw herbal powder.

#### 2.4.2. Light Microscopy Images of Congo red and Iodine-KI Stained Samples

**Figure 3:**
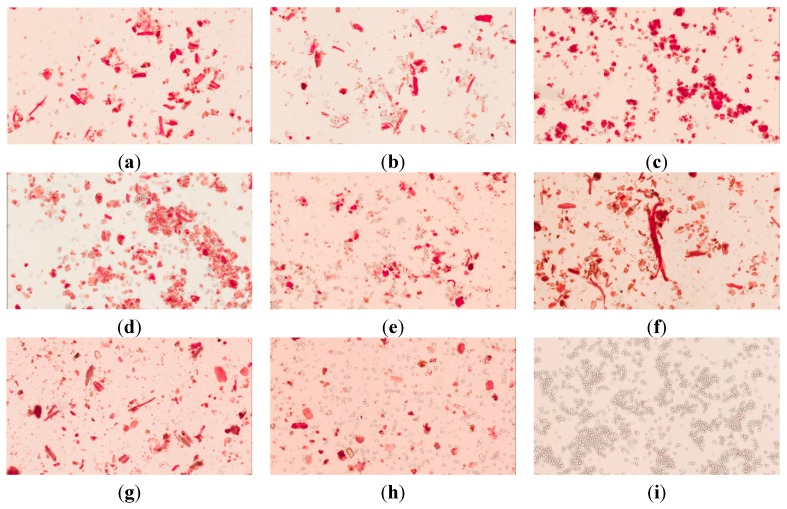
(**a**) Light microscopy images of Congo red stained samples: (**a**) brand A; (**b**) brand B; (**c**) brand C; (**d**) brand D; (**e**) brand E; (**f**) herbal powder; (**g**) herbal powder:brand A = 1:1, (**h**) corn starch:herbal powder = 1:1 and (**i**) corn starch.

To identify cellulose fibrils’ content, Congo red has been widely used in phytochemistry [19]. Congo red shows a preferred orientation of its average dipole moment along the long axis of the fibrils [20]. By binding the β1-4 glucan, the affinity of Congo red to fiber is very strong. The sample that contained cellulose fiber was red-stained, whereas corn starch (Figure 3i) was not stained by Congo red. The herbal powder was irregular and filamentous. The pharmaceutical powder samples of brands A–E exhibit rod-like shapes, and the granules were more uniformly distributed. These results demonstrated that grinder-crushed herbal powder had been added to the final pharmaceutical products.

**Figure 4:**
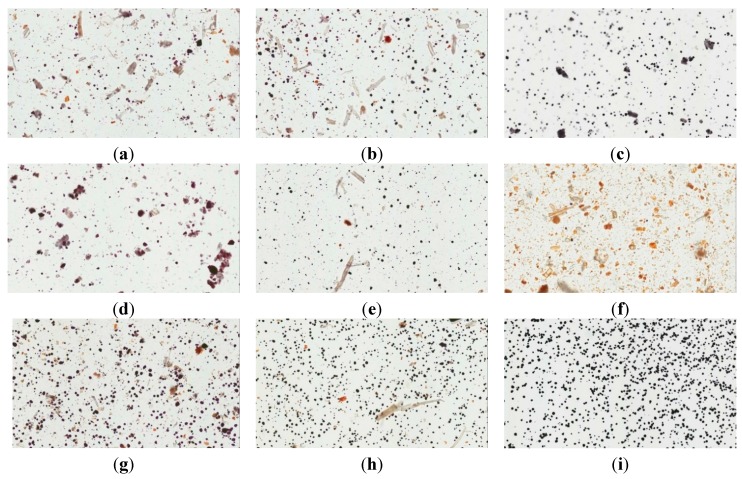
Light microscopy images of iodine-KI stained samples: (**a**) brand A; (**b**) brand B; (**c**) brand C; (**d**) brand D; (**e**) brand E; (**f**) herbal powder; (**g**) herbal powder:brand A = 1:1; (**h**) corn starch:herbal powder = 1:1 and (**i**) corn starch.

To identify the starch content in the pharmaceutical products, the iodine-potassium iodine staining method was applied. The proportion of iodide is important because iodine plays a role in the target penetration of iodine from 10% iodine-potassium iodide solution [21]. The raw herbal powders are mainly composed of rootstock botanic fibers. The endogenic starches in the root and rhizome gave rise to different dye concentrations. However, the starch and some adjuvants added as additives during the manufacturing processes were significantly stained by the iodine-potassium iodide solution (Figure 4). Corn starch, which was totally stained (Figure 4i), was used as a positive control. Contributing to the gelatinization of the prilling procedure, starch granule particles have different sizes in different brands of pharmaceutical powder (Figure 4a–e). The light microscope studies demonstrated that some of the crude fibers were isolated and some were merged into the gelatinized complex in those pharmaceutical samples. Possible additives to the pharmaceutical powder, such as starch and crude fiber, can be distinguished using our method, and our data provide a significant index of HGW purity.

#### 2.4.3. Solubility and Swelling Experiment

To examine the physical properties of starch in pharmaceutical products, the solubility and swelling were examined using a heated water bath. The results show that the solubilities of pharmaceutical products for brands A–E were 35.21%–36.86%, 41.26%–44.61%, 36.43%–38.21%, 41.63%–44.98%, and 31.48%–35.64%, respectively (Table 4). Meanwhile, the swelling of the pharmaceutical products for brands A–E were 5.05%–5.73%, 3.93%–4.72%, 5.85%–6.93%, 4.57%–5.16%, and 4.20%–6.04%, respectively (Table 5).

**Table 4:** Solubility analysis of the herbal pharmaceutical products at different temperatures.

Sample Brand	55 °C	65 °C	75 °C	85 °C	95 °C
**A**	38.73 ± 2.77	38.84 ± 0.72	40.82 ± 3.60	42.59 ± 0.19	45.74 ± 2.07
**B**	45.95 ± 4.84	47.78 ± 2.61	46.18 ±0.62	50.13 ± 0.94	52.97 ± 2.93
**C**	40.97 ± 2.58	42.19 ± 1.47	43.62 ± 1.31	42.76 ± 1.32	48.81 ± 3.95
**D**	33.67 ± 2.31	35.20 ± 0.97	39.95 ± 2.94	38.77 ± 1.68	40.64 ± 3.89
**E**	39.51 ± 0.19	41.33 ± 3.22	42.51 ± 2.85	41.63 ± 5.62	45.49 ± 1.56

Each value is expressed as the mean ± S.E.M (*n* = 3).

**Table 5:** Swelling analysis of the herbal pharmaceutical products at different temperatures.

Sample Brand	55 °C	65 °C	75 °C	85 °C	95 °C
**A**	4.99 ± 0.71	6.26 ± 0.31	6.22 ± 0.82	7.41 ± 0.28	6.86 ± 1.14
**B**	5.96 ± 1.64	7.44 ± 1.38	8.83 ± 0.21	8.79 ± 1.44	6.69 ± 1.19
**C**	6.43 ± 0.57	6.15 ± 1.09	8.93 ± 0.35	7.45 ± 1.99	4.74 ± 0.71
**D**	4.78 ± 0.31	5.37 ± 0.71	5.42 ± 1.58	5.59 ± 0.53	7.71 ± 1.54
**E**	4.04 ± 0.41	4.57 ± 0.89	5.81 ± 0.43	5.25 ± 0.85	6.60 ± 0.60

Each value is expressed as the mean ± S.E.M (*n* = 3).

Previous studies reported that the water solubility and swelling of starch increase with increasing temperature [22]. The starch granular swelling is a primary property of amylopectin, for example, and a waxy starch seen in our study is different from a normal starch [23]. However, the solubility and swelling changes of the five manufactured products only occurs within a limited range. Consequently, we suggest that the solubility and swelling data provide indirect information on starch content in the pharmaceutical products.

#### 2.4.4. Crude Fiber Content

To evaluate the crude raw herbal powder content in the industrial pharmaceutical herbal products, the crude fiber content (%) was examined. The results demonstrated that the crude fiber contents for the whole raw herbal powder, the herbs + starch (1:1, *w*/*w*) and pure corn starch were 20.65% ± 0.24%, 12.09% ± 0.44%, and 0.16% ± 0.02%, respectively. The raw herbal powders were all from the plant crush, and they represent the maximum cellulose fiber content in the prescription. The corn starch showed very little crude fiber in this examination. The mixture of half herbs and half starch contained half the fiber compared with the raw herbal powder. The crude fiber contents for the pharmaceutical product for brands A–E were 12.98% ± 1.27%, 5.95% ± 0.28%, 18.46% ± 0.01%, 6.76% ± 0.30%, and 9.22% ± 0.34%, respectively. These results demonstrate that all the tested pharmaceutical herbal products contained crude fiber to some extent. Among the different products, brand C contained the highest level of crude fiber.

## 3. Experimental Section

### 3.1. Reagents and Materials

Astragaloside I, astragaloside IV, formononetin and paeoniflorin were purchased from Tauto Biotech Co. Ltd. (Shanghai, China). The 6-gingerol and cinnamic acid were purchased from Sigma-Aldrich Chemicals (St. Louis, MO, USA). Ammonium acetate and methanol were obtained from E. Merck (Darmstadt, Germany). Deionized water (Millipore, Bedford, MA, USA) was used throughout the experiment. The HQW herbal pharmaceutical products were obtained from five major pharmaceutical manufacturers in Taiwan, including Sun Ten Pharmaceutical Co., Ltd. (Taipei, Taiwan), Kaiser Pharmaceutical Co., Ltd. (Tainan, Taiwan), Chuang-Song-Zong Pharmaceutical Co., Ltd. (Kaohsiung, Taiwan), Koda Pharmaceutical Co., Ltd. (Taoyung, Taiwan), and Sheng Chang Pharmaceutical Co., Ltd. (Taipei, Taiwan). The names of the five manufacturers are coded in this study to preserve the commercial confidentiality of the analysis results.

### 3.2. Preparation of Herbal Extraction

The original five dry HGW herb compositions were purchased from an herbal drugstore in Taipei and extracted at the National Research Institute of Chinese Medicine, Taipei, Taiwan. The preparation procedures for the herbal formula have been described in previous reports [24,25]. The mixed crude dry herbs of HGW contained *Astragalus membranaceus* (102 g), *Cinnamomum cassia* (51 g), *Paeonia lactiflora* (51 g), *Zingiber officinale* (51 g) and *Ziziphus jujube* (36 g) that were weighed in the proportion of 10:5:5:5:3. The admixture of crude herbs was extracted twice using a reflux apparatus with a 2000 mL ethanol-water (50:50, *v*/*v*) solution at 70 °C for 5 h. The collected extracts were combined after filtration and concentrated by a rotary evaporator to remove the water. The process finally yielded 83.65 g of the lyophilized HGW powder.

### 3.3. UPLC-ESI-MS/MS System and Experimental Condition

The LC-MS/MS analysis was executed on a Shimadzu LCMS-8030 triple quadrupole mass spectrometer equipped with an electrospray ionization interface and was coupled to the UFLC system (Shimadzu, Kyoto, Japan). The chromatographic separation of analytes was carried out using a C_18_ column (100 × 2.1 mm, particle size 1.7 μm, Waters, Dublin, Ireland). The analytes were eluted with a mobile phase consisting of 5 mM ammonium acetate (mobile phase A) and 100% methanol with 0.05% formic acid (mobile phase B), pumped at a flow rate of 0.2 mL/min. The gradient started at 30% mobile phase B for a minute and then was linearly increased to 90% B over 3 min. Subsequently, the eluent composition was maintained for 3 min before it was decreased to 30% mobile phase B. It was returned to its initial conditions in 1 min and then equilibrated 3 min at the end. The electrospray ion source was operated in positive and negative ionization modes for the analysis. The following instrument conditions were used: interface voltage, 4.5 kV; desolvation line temperature, 250 °C; heat block temperature, 400 °C; desolvation gas, nitrogen; desolvation gas flow rate, 3 L/min; drying gas, nitrogen; drying gas flow rate, 17 L/min; collision gas, argon; and collision gas pressure, 230 kPa.

### 3.4. Preparation of the Sample

Stock solutions of the analytes were dissolved in 100% methanol to a final concentration of 1 mg/mL and stored at −20 °C. To prepare the calibration standard solutions, a series of final concentrations were created by diluting with 50% (*v*/*v*) methanol as follows: 5, 10, 25, 50, 100, 250, 500 and 1000 ng/mL. All the calibration standard solutions were kept at 4 °C and were brought to room temperature for analysis. The sample was prepared by using 0.4 g of Chinese pharmaceutical herbal powder dissolved in 100 mL of methanol, followed by extraction in an ultrasonic water bath for 15 min, and centrifugation at 13,000 rpm for 10 min at 4 °C. The supernatant was filtered through a 0.22 μm syringe filter. This was diluted to the proper concentration, and then 10 μL was injected into the LC-MS/MS for analysis. The determination of all marker compounds was converted into the real content by: (mg/g) = [determined concentration (ng/mL)/concentration of sample (4 mg/mL)] × dilution ratio.

### 3.5. Preparation of the Samples

Good fitting coefficients (*r*^2^) for the calibration curves were determined by plotting the peak area ratio, creating a linear plot for the six compounds. The limit of detection (LOD) and quantification (LOQ) were determined at signal-to-noise ratios (S/N) of 3:1 and 10:1, respectively. The accuracy and precision were required to be within ±15%, and the LOQ values were within ±20%. The intra-day and inter-day variations were evaluated using six replicates. The accuracy was estimated by the nominal concentration (Cnom) and the mean value of the observed concentrations (Cobs) as follows: accuracy (%, bias) = [(Cobs − Cnom)/Cnom] × 100. The precision, relative standard deviation (RSD), was calculated from the observed concentrations as follows: RSD (%) = (standard deviation/Cobs) × 100.

### 3.6. Physical Examination of Additives for Raw Herbal Powder

#### 3.6.1. Scanning Electron Microscope

The commercial HGW herbal pharmaceutical products were purchased from five different pharmaceutical manufacturers in Taiwan. The outer appearance of the herbal pharmaceutical powders was observed through a scanning electron microscope (JSM-5300, Jeol Ltd., Tokyo, Japan). The crude HGW herbs were obtained from a traditional herbal shop in Taipei and prepared as a raw herbal powder at the National Research Institute of Chinese Medicine, Taipei, Taiwan. The crude herbs were milled using a hammer mill (Hung Chuan RT-04, Taipei, Taiwan) and passed through a 60-mesh sieve. Food-grade corn starch was purchased from Sun Right Co., Ltd. (New Taipei City, Taiwan) and passed through a 60-mesh sieve. The lab-made herbal powder was dried for 24 h in an oven (DO45, DENGYNG Instruments Co., Ltd., New Taipei City, Taiwan) at 40 °C and then placed on an aluminum support with glue or double-sided adhesive tape and coated with gold using a gold sputter module for 90 s in a high vacuum evaporator (JFC-1200 Ion Sputterer, Jeol Ltd.). The samples were subsequently analyzed under a scanning electron microscope. Then, the raw herbal fine powder and corn starch were examined in the same manner as just described.

#### 3.6.2. Light Microscopy Images of Congo Red and Iodine-KI Stained Samples

Light microscopy images were acquired by light microscopy (Aperio Scanscope CS system, Aperio, CA, USA) with Congo red staining to qualitatively evaluate the cellulose fibers in the Chinese pharmaceutical herbal products. The samples (3%) were prepared with glycerol/20% ethanol (1:1, v/v) on the microslide. Finally, the samples were stained with 0.05% Congo red and then viewed under a microscope at 100-fold magnification. The transferred Congo red-to-10% iodine-potassium iodide solution was used to qualify the gelatinized starch and treated samples in the same manner.

#### 3.6.3. Solubility and Swelling Experiments

The water solubility and swelling index experiments were modified from the previous report [22]. The individual various brands of herbal pharmaceutical powder (0.45 g) and deionized water (30 g, 1.5%, *w*/*w*) were heated in a water bath (BH-230D, YIN DER Instruments Co., Ltd., New Taipei, Taiwan) at 55, 65, 75, 85, and 95 °C for 1 h. After heating, the samples were cooled to room temperature and centrifuged at 8000 *g* for 20 min to separate the supernatant and sediment precipitations. The solute was put into a dry oven at 100 °C, and the resultant weight of residue is symbolized as W1, and the weight of the sediment in the centrifuge tube is symbolized as W2. The solubility was calculated using the formula of solubility = (W1/pharmaceutical herbal powder weight) × 100%; swelling = W2/[pharmaceutical herbal powder weight × (1 − solubility/100)].

#### 3.6.4. Crude Fiber Analysis

The pharmaceutical herbal powder (2 g) was boiled in 1.25% H_2_SO_4_ (200 mL) for 30 min and then washed with hot distilled water and filtered using a suction apparatus. Then, 1.25% H_2_SO_4_ (200 mL) was added into a solution of 1.25% NaOH, and the sample was treated in the same way. The residue was dried in an oven at 100 °C for 24 h to achieve a constant weight and then ignited in a muffle furnace (DF202, DENGYNG Instruments Co., Ltd.) at 550–600 °C for 5–6 h until grey ash was obtained. The residue was cooled in a desiccator and weighed. The percentage of crude fiber (%) was calculated based on the following formula: crude fiber (%) = 100 × (constant weight of residue – weight of ash)/weight of sample.

## 4. Conclusions

The study developed a rapid, selective and validated liquid chromatography-tandem electrospray ionization mass spectrometry method to determine the biomarkers astragaloside I, astragaloside IV, formononetin, cinnamic acid, paeoniflorin and gingerol in commercial Huangqi-Guizhi-Wuwu products. Additionally a rigorous examination to evaluate the quality of the herbal products was performed. The results demonstrate deviations in biomarker content and physical properties across different brands, suggesting the importance of the quality assessment for those commercial manufacturers.
